# Big Data Analysis, Design, Effect Fabrication, and Properties Analysis of SiO_2_/Cr/SiO_2_ Colored Solar Selective Absorbers with High PTCE and Chromaticity for Building Applications

**DOI:** 10.3390/ma17235810

**Published:** 2024-11-27

**Authors:** Fu-Der Lai, Yen-Ting Lai, Chang-Song Chen

**Affiliations:** 1 Institute of Photonics Engineering, National Kaohsiung University of Science and Technology, Kaohsiung 82445, Taiwan; chen1chang1song@gmail.com; 2 Department of Information and Computer Engineering, Chung Yuan Christian University, Taoyuan City 320314, Taiwan; jeremy10350206@gmail.com

**Keywords:** chromaticity, PTCE, colored solar selective absorber, big data analysis

## Abstract

In today’s era of rapid computing, advanced big data analytics enables precise results and efficient trend analysis. By leveraging these tools, the influence of various film thicknesses of Colored Solar Selective Absorbers (CSSAs) on solar absorption efficiency (α_s_) and chromaticity was investigated. A clear and visually informative Chromaticity Coordinate Distribution (CCD) versus α_s_ diagram was constructed within the CIE xy chromaticity diagram, establishing a correlation between chromaticity and α_s_. Photo-Thermal Conversion Efficiency (PTCE) ≈ α_s_ − 2% when α_s_ ≥ 90%. Subsequently, utilizing colored CCD-α_s_ diagrams, seven SiO_2_/Cr/SiO_2_ CSSAs, each with unique colors and α_s_, were designed, fabricated, and subjected to an analysis of their optical and material properties. We explored the influence of oxygen atom infiltration into the CSSA, leading to the oxidation of the Cr layer and consequent alterations in CSSA properties. Additionally, this study delved into analyzing the effect of substrate surface roughness on the oxidation resistance, α_s_, color, and corrosion resistance of CSSAs.

## 1. Introduction

The results of simulations for the optical properties of thin films often align closely with actual coating results [1], highlighting the importance of thin film optical simulations in coating technology. A comprehensive understanding of the properties of multi-layer optical coatings, such as the solar absorption efficiency (α_s_), Photo-Thermal Conversion Efficiency (PTCE), and color in Colored Solar Selective Absorbers (CSSAs), is unachievable without detailed simulations before their coatings. These simulations could quickly provide designers with crucial feedback on whether the film adheres to the set optical specifications, making thin film optical simulations an essential work of the coating process’s preparatory phase.

In the current era of rapid computing and advanced big data analytics, conducting spectral analysis (wavelength range: 250 to 2000 nm) of Dielectric-Metal-Dielectric (DMD) films with large data sets (exceeding one million sets) has become more feasible and important. Comprehensive and precise optical simulations of thin films are essential for understanding how film thickness variations affect the color alterations and α_s_ (or PTCEs). After completing the simulation, it is crucial to conduct an in-depth analysis of the big data to convert the simulation data into actionable insights. Big data analysis offers comprehensive and detailed results, and presenting these results graphically enables users to interpret and apply the findings easily. Therefore, it is essential to use big data analysis results to develop an intuitive, simple, and clear diagram that illustrates the optical properties of optical films. This visual representation will help users understand the relationship between chromaticity and high α_s_, illustrating the range of optical properties available, such as identifying the optimal percentage of αₛ for each color and the colors that meet the criteria of 92 ≤ αₛ < 93%. Additionally, when specific optical property requirements, such as αₛ > 96% and yellow color, are provided, the thickness of each layer of CSSA can be quickly determined by using the graph results and inputting these requirements in the simulated data file.

Integrating solar thermal energy into house design is crucial for saving energy. Solar air heating systems utilize solar energy to heat or ventilate indoor spaces, applicable to both new and renovated buildings [2]. This involves incorporating large-area solar selective absorbers (SSAs) into various building parts. For example, SSAs can be integrated into exterior walls to convert solar energy into thermal energy, which is then transmitted for indoor use [2,3]. Additionally, SSAs can be incorporated into the dome of the roof or stairwell enclosure [4], where they convert sunlight into heat. This heat increases the volume and decreases the density of the nearby air, causing it to rise and create convection currents. These currents draw in cooler air from the basement or outside, thereby enhancing natural ventilation and reducing indoor temperature. The natural ventilation system/solar chimney [4], exemplified in the Faculty of Environmental Engineering at the University of Kitakyushu, Japan, demonstrates that combining natural exhaust through the solar chimney with introducing fresh air via an underground cool pit reduces energy consumption for air conditioning. To maximize the absorption of solar energy, the surface of the collector absorber is currently black or blue [2,3,4,5,6,7,8,9,10,11,12,13,14,15,16,17,18,19,20,21,22,23,24,25,26,27,28,29,30,31,32,33,34]. However, using black or blue SSAs on building roofs and facades limits their aesthetic appeal, making their integration into the buildings more difficult. Survey results indicate that 85% of architects prefer colored SSAs [CSSAs] rather than black or blue ones. Developing a Colored Solar Selective Absorber layer material would allow more diverse simulations for easier integration into the building plan [32]. The better the PTCE of the solar selective absorber layer is, the better the performance of the solar heat energy collector.

Multilayer films [18,19,20,21,22,23,24,25,26,27,28] for SSA applications have been studied, but only some multilayer films for CSSAs [1,35,36,37,38,39,40,41,42,43,44,45,46] have been developed. The multilayer coatings of MoNbHfZrTi [35], Al_2_O_3_/Ti/Al_2_O_3_ [36], Cu/Ti/SiO_2_/Ti/TiO_2_/SiO_2_ [37], Cu/TiN_x_O_y_/TiO_2_/Si_3_N_4_/SiO_2_ [38], and TiO_2_/Si_3_N_4_/SiO_2_ [39], the colored absorbers with a TiN*_x_*O*_y_* absorbing layer [39] and the double cermet layer Al–AlN solar absorber coating [40], and a five-layered quarter-wave stack (optical model: air//HLHLH//glass//HLHLH//air) were elaborately simulated for the Colored Solar Selective Absorbers. The relationship between the reflection angle and optical reflectance spectrum was discussed by A. Schuler et al. [42]. However, α_s_ value and chromaticity will change with changes in CSSA film material or film thickness. Graphically presenting the effect of film thickness on simulation results will simplify comprehension for researchers and design analysts. Contour maps depicting the relationship between α_s_ and layer thickness for Al_2_O_3_/Pt/Al_2_O_3_ [1] and SiO_2_/Cr/SiO_2_/Cr [45] SSAs, as well as the chromaticity (x,y) diagrams depicting the relationship between chromaticity and layer thickness for Al_2_O_3_/Ti/Al_2_O_3_ CSSAs [36], have been published in our previous research. But, to easily achieve the precise design and fabrication of a CSSA with a specific α_s_ value and hue, it is imperative to establish a visual graphical relationship diagram correlating chromaticity with α_s_ values, providing researchers and design analysts with an easily comprehensible tool for investigating and fabricating Colored Solar Selective Absorbers. However, no reports in the literature graphically visualize the relationships between chromaticity and α_s_ value in a CIE xy chromaticity diagram for CSSAs. Therefore, a colored diagram is expected to easily visualize the effects of layer thicknesses in the different layers of CSSAs on the α_s_ value and chromaticity in a CIE xy diagram.

The surface roughness of the substrate will affect the performance of thin films. For example, a TiN coating applied on a substrate with a roughness of 0.4 µm demonstrates a reduced coefficient of friction [47]. The adhesion strength exhibits an initial increase followed by a subsequent decline with increasing surface roughness of the substrate [48]. My research group also investigated the influence of substrate surface roughness on the characteristics of CrN hard films [49]. CSSAs are intended for integration into the building exterior, so their properties are subject to environmental influences. Consequently, this study will also investigate the effect of substrate surface roughness on the oxidation resistance, PTCE, color, and corrosion resistance of CSSAs.

The characteristics of DMD films have been discussed in detail [50], and they have the advantages of excellent PTCE and easy design and production. Therefore, the films discussed in this article are composed of DMD structures. SiO_2_/Cr/SiO_2_ DMD structures exhibit excellent absorption efficiency in the visible light region, making them widely used in various applications such as high-contrast displays [51] and CSSAs [45].

In this work, using big data analysis, a series of colored CIE xy chromaticity diagrams, which clearly and visually depict the relationship between α_s_ and chromaticity, are constructed and analyzed. Seven CSSAs meeting the requirements of each color, different α_s_ values, and other Chromium (Cr) layer thicknesses are designed and fabricated, and their properties are measured. The effect of substrate surface roughness on the PTCE, oxidation resistance, and corrosion resistance is also studied. There are four main aspects discussed in this study:

For analysis via thin film optical simulation for CSSAs,

*1.*  *Study on graphing the colored chromaticity coordinate distribution (CCD)-α_s_ diagram in the CIE xy chromaticity diagram and analyzing the relationship between α_s_ values and CCD;*

(Colored CCD-α_s_ diagram: The data were plotted in the CIE xy chromaticity diagram to visualize the CCD across the simulated CSSA set and the data with different α_s_ values are represented by different colors. The color diagram visually represents the relationship between chromaticity and α_s_ value).

For design and fabrication via colored CCD-α_s_ diagrams,

*2.1.*  *Study on the design, fabrication, and analysis of seven SiO_2_/Cr/SiO_2_ CSSAs using colored CCD-α_s_ diagrams* *;*

For properties analyses,

*2.2.*  *Study on the impact of oxygen atom penetration on the crystallographic structure of CSSA during annealing;*
*3.*  *Study on the effect of surface roughness on the properties of fabricated CSSAs;*
*4.*  *Finally, provide a detailed discussion and explanation of the relationship between experimental results and surface roughness or simulations.*

## 2. Experimental Details

### 2.1. Preparation of the SiO_2_/Cr/SiO_2_ Colored Solar Selective Absorbers

The substrate cleaning steps and substrate dimensions are the same as those in the previous literature [1,36]. The high-purity evaporation materials are 99.99% pure SiO_2_ and 99.99% pure Cr particles. When depositing SiO_2_ and Cr layers, O_2_⁺ and Ar⁺ ions sources are used, respectively. The deposition rates of SiO_2_ and Cr layers are, respectively, controlled to be 0.1 and 0.05 nm/sec. The SiO_2_/Cr/SiO_2_ CSSAs were deposited on the substrates, including mirror-finish Cr/Si wafer, Cr/mirror-like SS304 stainless steel, and sandblasted SS304 stainless steel. The SiO_2_ and Cr layers were deposited by an ion source-assisted electron beam evaporation system with a high-powered electron gun operating at 10 KW and an ion gun with a power output of 3 KW at a vacuum degree of up to 1 mPa.

### 2.2. Characterization of the Thin Films

The reflectance spectrum in the wavelength range of 250–2000 nm is measured by an optical spectrometer (V-670, ISN-723) with the integrating sphere. The refractive index *n* and extinction coefficient *k* of the SiO_2_ and Cr films are measured by the spectroscopic ellipsometer (J. A. Woollam, Lincoln, NE, USA, M-2000 DI). All color photos are taken by a Canon camera. X-ray diffraction is used to examine the crystallographic structures of the CSSAs. The cross-sectional image of the films is measured by field emission scanning electron microscopy. The surface roughness is measured by a α stepper (KLA Tencor Alpha-Step IQ). For electrochemical potentiodynamic measurement, the experimental arrangement uses a three-electrode system. Scans are initiated by lowering the corrosion potential of the specimen from a preset value of −1.0 V (vs. SCE) to 1.0 V at a rate of 0.25 mVs^−1^. All experiments are conducted in 1N NaCl solution at a room temperature of about 25 ℃. The reflectance spectrum in the wavelength range of 2500–20,000 nm is measured by an FTIR with the integrating sphere.

### 2.3. Optical Properties of Simulation for CSSAs Using the CUDA C Parallel Computation Technology

Due to the detailed and accurate nature of the thin film optical simulations conducted, a significant volume of data was necessary to generate one specific diagram: the CIE xy chromaticity diagram. To handle this extensive data processing efficiently, CUDA C’s rapid parallel computing technology was utilized, accelerating the simulation process considerably. For the analysis, specialized software equipped with three key functionalities was employed, encompassing thin film optical simulation, α analysis, and analysis of color distribution coordinates.

#### 2.3.1. Thin Film Optical Simulation for the SiO_2_/Cr/SiO_2_ CSSA

A Cr layer approximately 200 nm thick serves both as an adhesion layer and the substrate.

For a multilayer film comprising m layers, the characteristic matrix at normal incidence is of the form [10,45]

(1) $$\left[\begin{array}{c} B\\ C \end{array}\right]=\prod_{r=1}^{m}\left[\begin{array}{cc} cos\alpha_{r} & \frac{isin\alpha_{r}}{N_{r}}\\ N_{r}sin\alpha_{r} & cos\alpha_{r} \end{array}\right]\left[\begin{array}{c} 1\\ N_{s} \end{array}\right],$$

(2) $$N_{r}=n_{r}-{ik}_{r},$$

(3) $$\alpha_{r}=\frac{2\pi }{\lambda }N_{r}d_{r}$$

where $N_{r}$ represents the optical constants of layer *r*; $n_{r}$ is the refractive index of layer *r*; $k_{r}$ is the extinction coefficient of layer *r*; $d_{r}$ is the thickness of layer *r*; and $N_{s}$ is the optical constants of the *Cr* substrate.

The reflectance R is calculated as follows [6]:

(4) $$R=\left(\frac{\eta_{0}B-C}{\eta_{0}B+C}\right){\left(\frac{\eta_{0}B-C}{\eta_{0}B+C}\right)}^{*},$$

where the $\eta_{0}$ in air is set to be 1.

The optical reflectance of the SiO_2_/Cr/SiO_2_ multilayer films is simulated utilizing Equations (1)–(4), along with parameters including the refractive index ($n_{r}$), extinction coefficient ($k_{r}$), and layer thickness ($d_{r}$).

#### 2.3.2. Analysis of Solar Absorption Efficiency, Emissivity, and α_s_ for the SiO_2_/Cr/SiO_2_ CSSAs

Reflectance for each film at any one wavelength can be derived from Section 2.3.1. Subsequently, solar absorption efficiency (α_s_) is calculated by fitting this reflectance data to the spectral power density of the standard AM 1.5 solar spectrum, as referenced in [36].

(5) $$\alpha_{s}=\frac{\int_{0.25}^{2.0}(1-R(\lambda ))\cdot SD(\lambda )d\lambda }{\int_{0.25}^{2.0}SD(\lambda )d\lambda }$$

where *R*(*λ*) and *SD*(*λ*) are, respectively, the measured reflectance and the spectral power density of AM1.5 at the wavelength of *λ*.

Subsequently, emissivity (ε) is calculated by fitting the reflectance data to the spectral power density of the thermal radiation, as referenced in [36].

(6) $$\varepsilon =\frac{\int_{2.5}^{25}(1-R(\lambda ))\cdot \mathrm{TE}(\lambda ,T)d\lambda }{\int_{2.5}^{25}\mathrm{TE}(\lambda ,T)d\lambda }$$

(7) $$\mathrm{PTCE}=\alpha_{s}-\varepsilon \frac{\left(\sigma T^{4}\right)}{\int_{0.25}^{2.0}SD(\lambda )d\lambda }$$

where *R*(*λ*) and TE(*λ*) are, respectively, the measured reflectance and the thermal radiation spectral power density at the film’s temperature T and wavelength *λ*. σT^4^ is the thermal radiation power at the film’s temperature T. σ is the Stefan–Boltzmann constant (5.67 × 10−8 W⋅m^−2^⋅K^−4^).

The mathematical expression for TE(*λ*) of a blackbody at a specific wavelength *λ* and temperature T is given by Planck’s law:

(8) $$TE\left(\lambda ,T\right)=\frac{2hc^{2}}{\lambda^{5}}\frac{1}{e^{\frac{hc}{\lambda kT}}-1}$$

where *c*, *h*, and *k* are the speed of light (3 × 10^8^ m/s), Planck’s constant (6.626 × 10^−34^ J∙s), and Boltzman’s constant (1.38 × 10^−23^ J/K), respectively. T is the absolute temperature in Kelvin. *λ* is the wavelength in meters.

**Figure 1:**
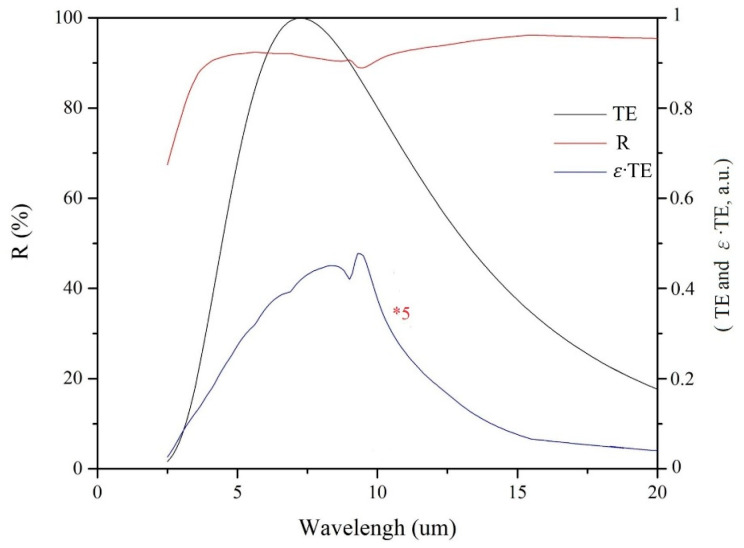
The IR reflectance (R), the thermal radiation spectral power density (TE), and the thermal radiation spectral power density (ε⋅TE) of the test piece (at 5× magnification) at 400 K.

Dan’s review article [50] on spectrally selective dielectric-metal-dielectric coatings provides a solar spectrum on the Earth’s surface and a temperature-dependent infrared radiation spectrum. When the film is at low temperatures (≤400 K), the intensity of solar energy at the Earth’s surface is significantly greater than the intensity of infrared radiation. When the solar absorption efficiency of a DMD film is greater than 90%, its emissivity is about 6% to 10%. Thus, the film’s infrared radiation intensity is less than 2% of the solar intensity (with a relatively small variation, indicating that the relationship between PTCE and αₛ approximates a linear relationship). For example, the absorption efficiency of all test pieces ranges from 90.5% to 93.2% [36], and their emissivity, as measured by FTIR, varies from 7.1% to 9.1%, with a typical range of 6% to 10%. Consequently, the film’s infrared radiation intensity is approximately 1.5% to 2.5% of the solar intensity at a film temperature of 400 K. In this study, a test piece with designed film thicknesses of 85/4.6/75 nm achieves a calculated α_s_ of 96.3%. Figure 1 illustrates the following: (1) the IR reflectance (R) is greater than 90% across most wavelengths, except between 9.2 and 9.8 microns, where it exceeds 89%; (2) the thermal radiation spectral power density (TE) at 400 K; and (3) the thermal radiation spectral power density (ε·TE) of the test piece (at 5× magnification). This test piece only has a calculated emissivity of 7.5%, which is also in the 6–10% range. Therefore, for calculation convenience, it can be considered as 2%.

Therefore, PTCE can be written approximately as

PTCE ≈ α_s_ − 2% (9)

#### 2.3.3. Analysis of Chromaticity for the SiO_2_/Cr/SiO_2_ CSSA

Tristimulus values for a color, characterized by its spectral power distribution *I*(*λ*) [40], are determined based on the standard observer as described by the following terms:

(10) $$X=\int_{380}^{780}I\left(\lambda \right)\bar{x}\left(\lambda \right)d\lambda ,$$

(11) $$Y=\int_{380}^{780}I\left(\lambda \right)\bar{y}\left(\lambda \right)d\lambda ,$$

(12) $$Z=\int_{380}^{780}I\left(\lambda \right)\bar{z}\left(\lambda \right)d\lambda ,$$

where $\bar{x}\left(\lambda \right),\bar{y}\left(\lambda \right),\bar{z}\left(\lambda \right)$ are the CIE 1931 color-matching functions; $I\left(\lambda \right)=D65\left(\lambda \right)R\left(\lambda \right).$ D65 is CIE Standard Illuminant D65.

The chromaticity of a color can be specified by the two derived parameters x and y as follows:

(13) $$x=\frac{X}{X+Y+Z},$$

(14) $$y=\frac{Y}{X+Y+Z},$$

where x and y are the values obtained by translating the measured optical reflectance spectra to CIE1931 chromaticity coordinates.

## 3. Results and Discussion

### 3.1. Big Data Analysis of Optical Thin Film for the SiO_2_/Cr/SiO_2_ CSSAs

#### 3.1.1. Simulation Settings and Optimal α_s_

**Figure 2:**
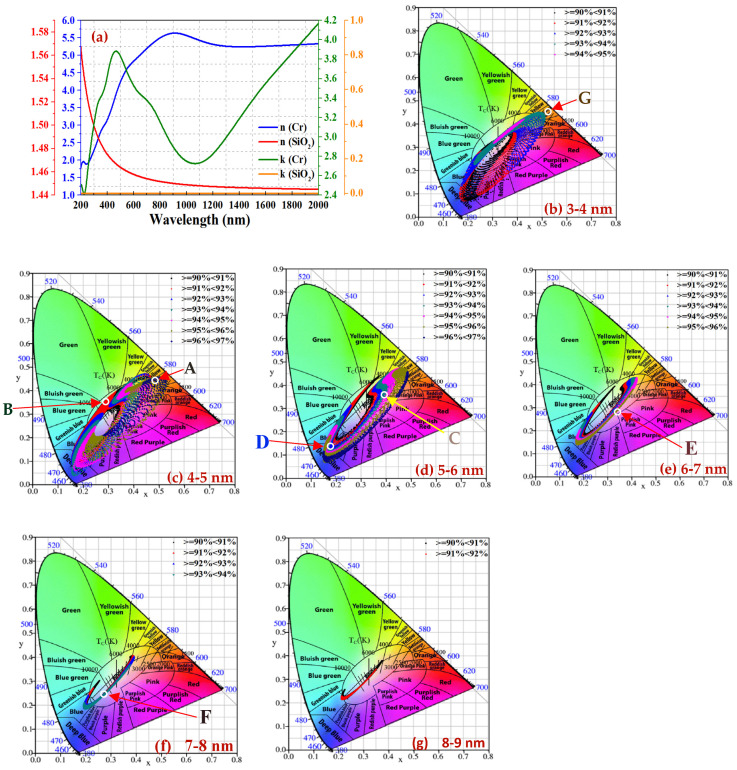
(**a**) The refractive index (n) and extinction coefficient (k) of both SiO_2_ and Cr layers and the CCD-α_s_ diagrams for the Cr layer thickness intervals ranging (**b**) 3–4, (**c**) 4–5, (**d**) 5–6, (**e**) 6–7, (**f**) 7–8, and (**g**) 8–9 nm. A to G are the designed and fabricated films. The colored CCD-α_s_ diagrams illustrate the relationship between chromaticity coordinates and α_s_ of CSSAs when the α_s_ is greater than 90%.

In the thin film optical simulation for SiO_2_/Cr/SiO_2_ CSSAs, the layer thickness parameters are set as follows: the top SiO_2_ layer ranges from 1 to 200 or 300 nm, with 1 nm intervals; the middle Cr layer from 1 to 15 nm, with 0.2 nm intervals; and the bottom SiO_2_ layer from 1 to 200 or 300 nm, also with 1 nm intervals. The simulation covers a wavelength range of 300 to 2000 nm. As depicted in Figure 2a, the refractive index (n) and extinction coefficient (k) of both SiO_2_ and Cr layers are determined using an ellipsometer. The substrates used are Cr/Si wafer, Cr/mirror-like SS304, and Cr/sandblasted SS304. On the substrate, SiO_2_/Cr/SiO_2_ layers are deposited.

The film thickness of the SiO_2_/Cr/SiO_2_ absorbers is defined as the top layer thickness/Cr layer thickness/bottom layer thickness. The unit of layer thickness is nm. From a total of 2,800,000 combinations, the optimal α_s_ is found in a film with thicknesses of 93 nm (top SiO_2_ layer), 5 nm (Cr layer), and 76 nm (bottom SiO_2_ layer) (i.e., 93/5/76), resulting in a maximum α_s_ of 96.5%. 

#### 3.1.2. Graphing Colored CCD-α_s_ Diagram in the CIE xy Chromaticity Diagram and Analyzing the Relationship Between α_s_ Value and CCD

In this section, utilizing the software of thin film optical simulation, α_s_ analysis, and chromaticity analysis and setting the α_s_ value greater than 90%, the film’s reflectance spectrum, α_s_, and chromaticity of CSSAs are determined and thus the relationship between the chromaticity coordinate and α_s_ is studied. This analysis enables mapping the film’s CCD, which is associated with the α_s_, onto the CIE xy chromaticity diagram. The data points with different α_s_ values are represented by different colors. This process effectively links the optical properties of the optical thin film, as revealed by the simulation, to its colorimetric representation, providing a comprehensive understanding of its chromatic distribution behavior in various simulated CSSAs.

Figure 2b–g present the colored CCD-α_s_ diagram, illustrating the relationship between chromaticity coordinates and α_s_ of CSSAs when the α_s_ is greater than 90%. Figure 2b displays a CCD-α_s_ diagram for a Cr layer thickness ranging from 3 nm (inclusive) to less than 4 nm, denoted as 3–4 nm. The subsequent Figure 2b–g display the CCD-α_s_ diagrams for Cr layer thickness intervals ranging from 3–4 nm to 8–9 nm. From Figure 2b, it can be found that the CCD-α_s_ diagram covers any one chromaticity coordinate. So, the CCD region (CCR) is very wide, including pink, orange, yellow, green, blue, and purple. But its green CCD Area (CCA) is the smallest compared to the other CCAs. Roughly speaking, the α_s_ within the same chromaticity area nearly remains the same. Compared to Figure 2b, Figure 2c also shows a broader CCR, with a notably larger green CCA. At the same α_s_, it encompasses a broader CCR; for instance, the CCR with α_s_ exceeding 96% spans various hues including pink, orange, yellow, green, blue, and purple. Notably, there is a broader region in the yellow-orange and orange regions. Additionally, across all diagrams from Figure 2b–g, the green CCA is the most expansive in Figure 2c.

Figure 2d illustrates that with an increase in the α_s_, the chromaticity coordinates region shifts further from the area of white light characterized by high-temperature white light (above 6000 K). But it encompasses the lower-temperature white light (below 4000 K) region. The yellow and orange CCAs are notably broader. Figure 2c,d indicate that the highest α_s_ can exceed 96%. The CCR shape in Figure 2e resembles that in Figure 2d but appears as a smaller version with a reduced CCA. Similarly, Figure 2f also mirrors the reduced version of Figure 2c,d, with an even smaller CCA, and the highest α_s_ is less than 94%. Figure 2d–f collectively show that as α_s_ increases, the chromaticity coordinate region moves from the high-temperature white light region. Figure 2g shows only a small CCR when α_s_ ≥ 90% and the highest α_s_ is less than 92%.

In summary, the distribution of α_s_ and chromaticity varies with the film thickness of the CSSAs, and the α_s_ and chromaticity of definite requirements can be readily obtained from the visualized colored CCD-α_s_ diagrams. Table 1 presents an analysis of the relationship between the α_s_ value and CCD in Figure 2b–g, along with the preparation of test samples.

### 3.2. Design and Fabrication of Seven CSSAs Utilizing Colored CCD-α_s_ Diagrams: Comprehensive Property Analysis Including Investigation into Oxygen Atom Penetration and Cr Layer Oxidation

#### 3.2.1. Design, Fabrication, and Analysis of Seven SiO_2_/Cr/SiO_2_ CSSAs Utilizing CCD-α_s_ Diagrams

Following the comprehensive thin film optical simulations and analyses, at least one film layer will be fabricated for each defined Cr layer thickness interval shown in Table 2, as depicted in Figure 2b–f. In Figure 2g, CCR is notably limited, and the peak α_s_ falls below 92%. Consequently, no film is fabricated within the Cr layer thickness range of 8–9 nm. Particularly for the Cr layer thickness intervals of 4–5 and 5–6 nm, as shown in Figure 2c,d, where the highest α_s_ value exceeds 96% and the areas with α_s_ greater than 90% are larger, two CSSAs in each interval will be fabricated. Furthermore, compared to other figures, Figure 2c is notable for its larger area, with the α_s_ exceeding 96%, where the yellowish-orange region is the most expansive.

**Figure 3:**
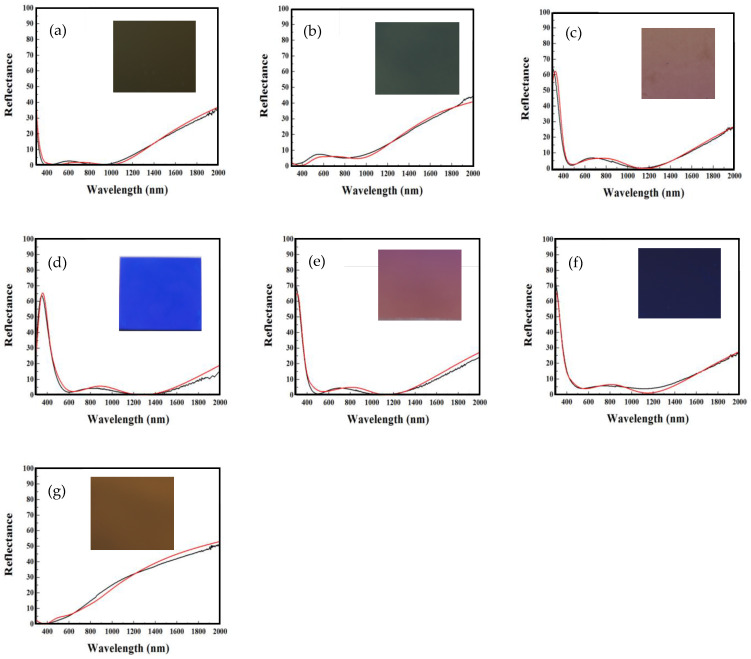
The reflected spectrum and photograph of the fabricated CSSAs for the layer thicknesses of (**a**) 85/4.8/76, (**b**) 62/4.8/61, (**c**) 90/5.2/101, (**d**) 119/5.8/108, (**e**) 97/6.2/86, (**f**) 95/7.2/88, and (**g**) 45/3.8/41 nm. The red and black lines are simulated and measured reflectance spectra of the designed film, respectively. The red and black lines are simulated and measured reflectance spectra of the designed film, respectively.

These analyses found that the simulation area is set to encompass the yellow and orange areas because the area with a α_s_ of more than 96% is wider. This approach enables a film to offer two key benefits: a golden hue and the highest α_s_. This simulated film with a thickness of 85/4.6/75 nm can achieve a α_s_ of 96.3% (its PTCE ≥ 94.3%). Figure 3a presents the reflectance spectrum of these simulated and fabricated films, along with a photograph of the fabricated film, which exhibits a dark gold color. The chromaticity coordinates of the fabricated film are located at point (A) in the yellowish-orange region of Figure 2c, with a film thickness of about 84.8/4.7/76.1 nm and a α_s_ of 96.2%. This verifies nearly the same results as that of the thin film optical simulations and analysis. Across all Cr layer thickness intervals, except for the 4–5 nm range, the area for green hue remains notably small. Consequently, a green SSA, as depicted at point (B) in Figure 2c, is designed and fabricated. This film, designed with a thickness of 62/4.8/61 nm and a α_s_ of 91.5% and fabricated with a thickness of about 62.1/4.8/61.5 nm and a α_s_ of 91.3%, exhibits a grass-green color. Its reflected spectrum and a photograph are displayed in Figure 3b.

Next, in Figure 2d, corresponding to a Cr film thickness of 5–6 nm, two CSSAs are designed in orange-pink and blue hues. In Figure 2d, the film thicknesses of the films at points (C) and (D) are designed to be 91/5.4/101 nm and 119/5.8/108 nm, respectively, with their α_s_ values being 94.4% and 92.2%, respectively. These films achieve α_s_ values of 94.5% and 92.1%, respectively. Their reflectance spectra and photos are illustrated in Figure 3c,d. Notably, Figure 3d shows that at wavelengths below 500 nm, reflectance increases with decreasing wavelength, especially in the blue light band where reflectance exceeds 10%, reaching over 35% at 400 nm, thus presenting a bright blue appearance.

In the 6–7 nm Cr film thickness interval, as illustrated in Figure 2e, within the purplish-pink chromaticity region, a film is analyzed and designed with a thickness of 96/6.4/86 nm, achieving an improved α_s_ of 95.1%. The fabricated film, positioned precisely at point (E) in Figure 2e, achieves an excellent α_s_ of 95.2%. The reflectance spectra of both the simulated and fabricated films, alongside the photo of the fabricated film, are presented in Figure 3e.

In the Cr layer thickness range of 7–8 nm, as shown in Figure 2f, the maximum observed α_s_ is below 94%. Therefore, a purple CSSA with a thickness configuration of 95/7.2/88 nm is designed and analyzed, labeled as F, achieving an α_s_ of 93.3%. The fabricated film achieves a α_s_ of 93.1%. The reflectance spectra for both simulated and fabricated films, along with the photo, are presented in Figure 3f. Finally, within the 3–4 nm Cr film thickness range depicted in Figure 2a, the color of the CSSA is designed to be a trendy color (earthy gold). Its chromaticity coordinates are located at point (G) in Figure 2a. This CSSA, with a minimal thickness of 46/3.8/43 nm (very thin first and third layers), still achieves a α_s_ greater than 80%. The α_s_ of the film fabricated according to this specification is 85.6%. The respective reflectance spectra and photos are shown in Figure 3g. This film demonstrates an increase of reflectance from 0% as the wavelength increases within the visible light spectrum (400 to 700 nm), and the reflectance exceeds 12% at 700 nm. And this fabricated film displays an earthy gold color.

**Figure 4:**
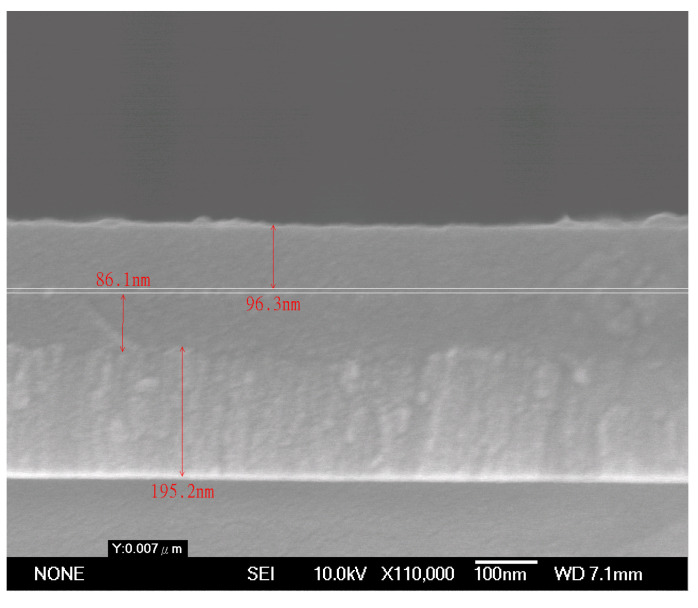
An SEM cross-sectional image of the CSSA.

Figure 4 displays the SEM cross-sectional image of the CSSA sample fabricated as shown in Figure 3e, illustrating layer thicknesses of approximately 96.3/6.4/86.1 nm. This is very close to the originally designed film thickness.

#### 3.2.2. Exploring the Impact of Oxygen Atom Penetration on the Crystallographic Structure of CSSA During Annealing

**Figure 5:**
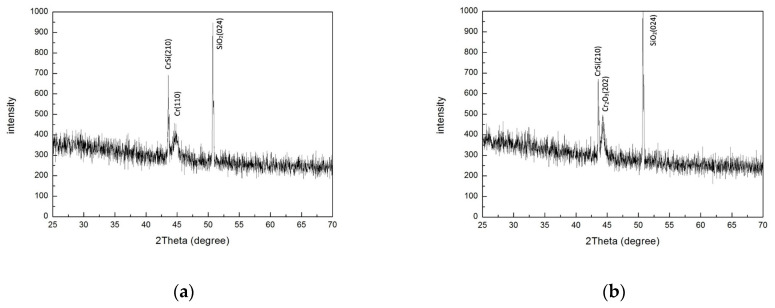
The XRD patterns, which depict the crystallographic structures of the CSSA (**a**) before and (**b**) after annealing.

Figure 5a,b present the XRD patterns illustrating the crystallographic structures of the CSSA before and after annealing, respectively. Before annealing, three distinct diffraction phases are observed: chromium silicide (CrSi) (210) [52], Cr (110), and SiO_2_ (024). After annealing, these phases transform into CrSi (210), Cr_2_O_3_ (202), and SiO_2_ (024). Notably, even before annealing, the CSSA exhibits a clear crystalline state, especially in the CrSi (210) phase. Driven by the impact of a high-energy ion-assisted source, this CrSi (210) phase results from the diffusion of Si atoms into the Cr layer and their subsequent reaction with Cr. After annealing in an air environment, it is observed that phase transition from Cr to Cr_2_O_3_ takes place. However, CrSi does not undergo a transition to chromium silicate (CrSiO). The phenomenon can be elucidated as follows: (1) Oxygen atoms or molecules permeate through the topmost layer of SiO_2_, subsequently interacting with Cr atoms within the Cr layer to form a compact layer of Cr_2_O_3_. (2) Oxygen atoms or molecules are hindered by the dense Cr_2_O_3_ layer, preventing further diffusion to the underlying layers, and thus inhibiting the oxidation of CrSi to CrSiO.

### 3.3. Effect of the Surface Roughness on Properties of the Fabricated CSSAs

**Figure 6:**
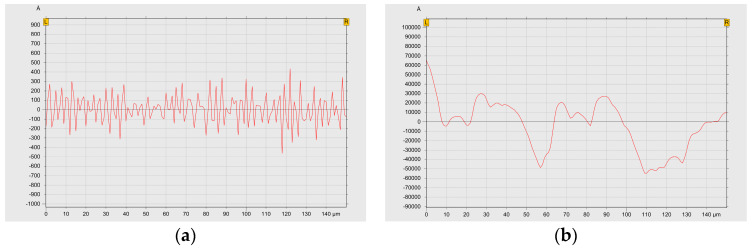
The RMS surface roughness of (**a**) the mirror-like SS304 and (**b**) sandblasted SS304.

The Si wafers used are of commercial-grade mirror surface quality, with a root mean square surface roughness (Rms) of less than 0.2 nm. The Rms of the mirror-like SS304 stainless steel and sandblasted SS304 stainless steel, measured using a α-stepper and illustrated in Figure 6, are 16 nm and 2642 nm, respectively. Mirror-like SS304 stainless steel is achieved through chemical mechanical planarization and electrolytic polishing. As shown in Figure 6a, there are no deep grooves on the surface of the mirror-like SS304 stainless steel. The sandblasted SS304 stainless steel is obtained by sandblasting the mirror-like SS304 stainless steel. Figure 6b shows that the distance between the grooves on the sandblasted surface is several microns, and the surface is uniformly rough. Cr films exhibit excellent adhesion properties with various substrates, making them a preferred choice for adhesion layers. Therefore, in this study, the absence of deep and narrow grooves on the surfaces of these three substrates allows Cr atoms to easily adhere uniformly, forming a complete and seamless adhesion layer.

**Figure 7:**
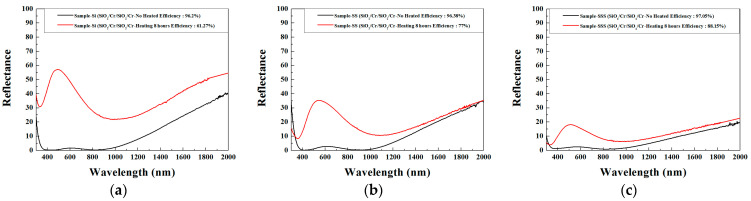
The reflectance spectra of the three CSSAs ((**a**) Y1, (**b**) Y2, and (**c**) Y3) both before and after annealing. The red and black lines are the reflectance spectra of these three CSSAs before and after annealing, respectively.

**Figure 8:**
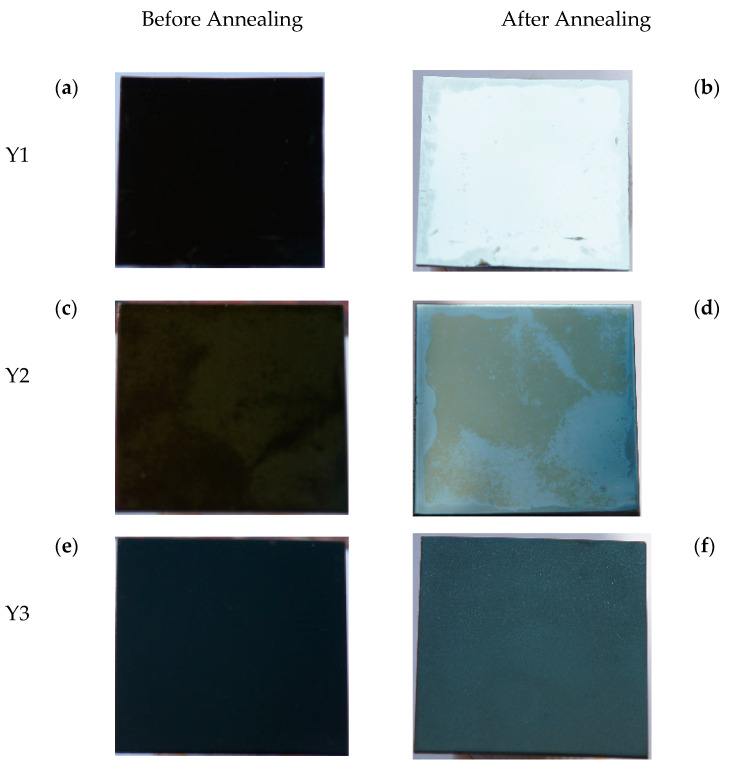
Photographs of Y1, Y2, and Y3, both before and after annealing.

SiO_2_/Cr/SiO_2_ solar selective absorbers deposited on substrates of Cr/Si wafer, Cr/mirror-like SS304, and Cr/sandblasted SS304 are designated Y1, Y2, and Y3, respectively. Figure 7a–c depict the reflectance spectra of these three CSSAs (Y1, Y2, and Y3) both before and after annealing for 8 h at 450 °C in an air environment. The black line represents the reflectance spectrum of the unannealed specimen, while the red line represents the reflectance spectrum of the annealed specimen. Initially, the α_s_s for Y1, Y2, and Y3 are 96.2%, 96.4%, and 97.1%, respectively, indicating that a rougher surface correlates with a higher α_s_. The rougher the surface, the more times the incident light will be reflected, resulting in multiple absorptions. Consequently, this leads to a higher α_s_. However, after annealing, the α_s_s decreased to 61.3%, 77.0%, and 88.2%, respectively, with a lesser reduction in α_s_ observed for rougher surfaces. This may be because a rougher surface increases the film layer’s adhesion, resulting in a denser film that is less susceptible to oxidation. Photographs of Y1, Y2, and Y3, both before and after annealing, are shown in Figure 8a–f. After annealing, Y1’s α_s_ declines from 96.2% to 61.3%, and its color shifts from dark gold to nearly white. The observed color shift is due to significant oxidation of the Cr layer, leading to a reduction in its absorbance. This results in the reflectance within the visible light spectrum surpassing 35% (as indicated by the red line in Figure 7a, giving the film an almost white appearance).

**Figure 9:**
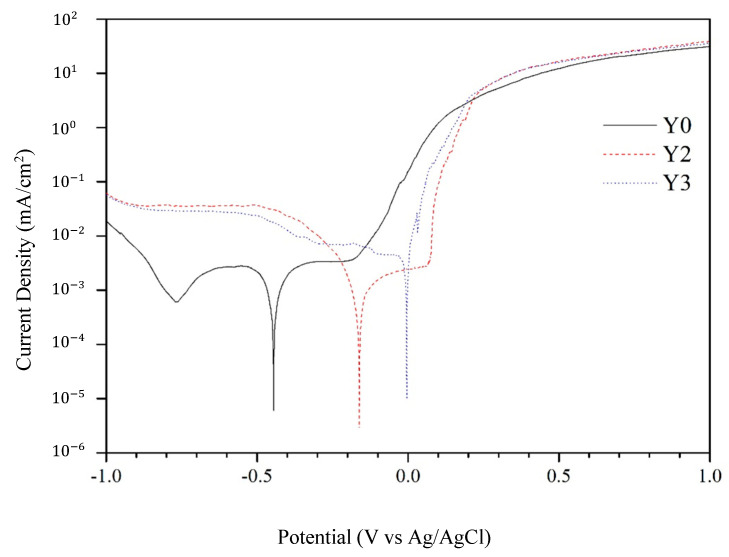
Evans diagrams for the three CSSAs (Y0, Y2, and Y3) in a 1 M NaCl solution.

Evans diagrams for three specimens (Y0, Y2, and Y3) in a 1 M NaCl solution are presented in Figure 9. Y0 is an uncoated mirror-like SS304. The values for corrosion potential (*ϕ*_corr_), corrosion current density (ⅈ_corr_), and corrosion rate are summarized in Table 3. SS304 is naturally corrosion-resistant, and both Y2 and Y3 exhibit enhanced corrosion resistance compared to SS304. It is observed that a rougher surface results in a lower corrosion current density and corrosion rate, and a higher corrosion potential, implying that a CSSA on a rougher surface more effectively reduces the material’s electrochemical activity in a corrosive environment.

### 3.4. Discussion and Explanation

Exploring the properties of films with millions of film thickness variations is impractical through experimental methods. However, using thin film optical simulation and analyses of α_s_ and chromaticity, the distribution of chromaticity coordinates based on α_s_ in the CIE xy chromaticity diagram can be easily explored and analyzed. This approach facilitates comprehension of the effects that variations in film thickness have on its α_s_ and chromaticity, thereby enhancing the exploration and analysis process. The conclusions drawn from Figure 2 are as follows: (1) Chromaticity and α_s_ demonstrate significant variations with changes in film thickness, as observed in Figure 2b–g. (2) Within a specified α_s_ range, such as 94% to 95%, the colored CCD-PTCE diagram depicts the colors and corresponding covered areas. (3) For a specific color requirement such as green, despite its smaller area, it can still determine the maximum attainable α_s_ and the corresponding film thickness. (4) For a desired color such as yellow-orange and a α_s_ exceeding 96%, the diagram illustrates the broadest area at Cr layer thicknesses ranging from 4 to 5 nm. Thus, akin to Figure 2, the colored CCD-α_s_ diagram facilitates the estimation of film thickness for specific α_s_ and color requirements.

The film thickness includes the thicknesses of the top layer, Cr layer, and bottom layer. Using software that includes thin film optical simulation and analysis tools for α_s_ and chromaticity, the α_s_ and chromaticity can be determined. The colored CCD-α_s_ diagram, as depicted in Figure 2, is constructed to visually demonstrate the relationship between the film’s chromaticity coordinates and α_s_. Using this diagram to design required CSSAs, firstly, the appropriate α_s_ and chromaticity coordinate region from Figure 2 are selected, followed by analyzing the film thickness to obtain a simulated film. Based on this approach, seven films are designed, chosen, and fabricated. Seven selected films are distributed across various colors and exhibit different α_s_ values. The optical properties of these fabricated films are consistent with the results obtained from the initial simulations and designs.

The specimens shown in Figure 3e, Figure 4, Figure 5, Figure 7a and Figure 8a are SiO_2_/Cr/SiO_2_ CSSAs deposited on Cr/Si wafer substrates. Influenced by a high-energy ion-assisted source, the unannealed CSSA exhibits a clear crystalline structure, especially in the CrSi (210) phase. The substrate transitions from a wafer substrate of Cr/Si to CrSi/Si. After annealing, the α_s_ of Y1 declines from 96.2% to 61.3%, with its color transitioning from dark gold to nearly white, as depicted in Figure 8a,b. As shown in Figure 5 and Figure 7a, the α_s_ decrease during annealing is attributed to the phase transformation from Cr to Cr_2_O_3_, indicating the oxidation of Cr to Cr_2_O_3_. However, CrSi does not oxidize to CrSiO, possibly because the oxidation of Cr in the Cr layer to dense Cr_2_O_3_ obstructs the passage of oxygen atoms or molecules. Inference suggests that oxygen atoms or molecules can initially oxidize the inner chromium layer through tiny pinholes in the surface layer in a vertical direction, followed by horizontal diffusion and continued oxidation within the layer.

It is observed that a rougher surface results in a smaller reduction in α_s_, suggesting that a rougher surface mitigates the oxidation of the chromium layer in the CSSA. This could be due to the presence of tiny pinholes in the top SiO_2_ layer facilitating Cr oxidation. At the same time, the rough surface’s undulations impede the continuous horizontal oxidation of oxygen atoms or molecules within the Cr layer. Pronounced surface undulations serve as a barrier to this horizontal oxidation.

The Evans diagrams also demonstrate that the rougher the surface, the greater the reduction in electrochemical activity.

Hence, a CSSA on a rougher surface exhibits higher α_s_, antioxidant, and anti-corrosion properties, thereby enhancing its overall performance.

The SiO_2_/Cr/SiO_2_ CSSA has a high PTCE of over 90%, reaching up to 94.5%, with a full range of color options and excellent weather resistance, making it ideal for any type of architectural application.

## 4. Conclusions

Thin film optical simulation plays a crucial role in optical thin film coating technology. However, there is currently a lack of literature providing simple and clear diagrams to analyze and visualize CSSA optical properties, specifically regarding the thickness of each layer to meet chromaticity and high α_s_ requirements. This study effectively explores and analyzes the impact of different film thicknesses on α_s_ and chromaticity, as demonstrated in the CIE xy diagram. A series of colored CCD-α_s_ diagrams were constructed and analyzed, revealing a broad color distribution for CSSAs with α_s_ exceeding 90%, encompassing all colors. Subsequently, leveraging the colored CCD-α_s_ diagrams, seven SiO_2_/Cr/SiO_2_ CSSAs were meticulously designed, fabricated, and subjected to optical and material property analysis. A film achieving an optimal α_s_ exceeding 96% and exhibiting a yellow-orange hue was successfully engineered, aligning with initial design expectations. The techniques and methodologies employed in this study enhance the precision and convenience of both research and manufacturing. They equip users, spanning from researchers to industrial design and production engineers, with a comprehensive set of tools for investigating and producing Colored Solar Selective Absorbers.

Moreover, this study analyzes the impact of oxygen atoms or molecules infiltrating CSSAs, leading to Cr layer oxidation and subsequent alterations in CSSA performance. Additionally, the effect of surface roughness on CSSAs is examined, revealing that rougher surfaces enhance α_s_, oxidation resistance, and corrosion resistance.

## Figures and Tables

**Table 1:** Analyzing the relationship between α_s_ value and CCD, and preparing the test pieces.

Figure Number	Thickness Range (X) of a Cr Layer (nm)	Chromaticity Coordinate Distribution Region	α_s_ Range (%)	Explanation of Chromaticity Coordinate Distribution and Preparation of Test Pieces
Figure 2b	3 ≤ X < 4	All Colors *	90 to 95	Its green CCD Area (CCA) is the smallest compared to other CCAs. Roughly speaking, α_s_ within the same chromaticity area nearly remains the same. (Prepare a test piece of G).
Figure 2c	4 ≤ X < 5	All Colors	90 to 97	The CCA with α_s_ exceeding 96% spans various hues including all colors. Notably, there is a broader region in the yellow-orange and orange regions. (Prepare a test piece of A). Across all diagrams from Figure 2b–g, the green CCA is most expansive in Figure 2c. (Prepare a test piece of B).
Figure 2d	5 ≤ X < 6	All Colors	90 to 97	With an increase in the α_s_, the chromaticity coordinate region shifts further from the white light region. The yellow and orange CCAs are notably broader. (Prepare two test pieces, C and D).
Figure 2e	6 ≤ X < 7	All Colors	90 to 96	The CCR shape in Figure 2e resembles that in Figure 2d but appears as a smaller version with a reduced CCA. (Prepare a test piece of E).
Figure 2f	7 ≤ X < 8	pink, orange, blue, and purple	90 to 94	Figure 2f also mirrors the reduced version of Figure 2c,d, with an even smaller CCA, and the highest α_s_ is less than 94%. (Prepare a test piece of F).
Figure 2g	8 ≤ X < 9	pink, blue, and purple	90 to 92	There is only a small CCR when α_s_ ≥ 90% and the highest α_s_ is less than 92%.

* “All colors” means complete coverage of the entire color gamut, including pink, orange, yellow, green, blue, and purple.

**Table 2:** Film thickness, PTCE, and color for designed and fabricated CSSAs.

Test Piece Encoding	In Figure	Designed Film Thickness (nm)	Reflectance Spectrum of Test Piece (Figure)	Calculated α_s_ (%)	Measured α_s_ (%)	Color
A	Figure 2b	85/4.6/75	Figure 3a	96.3	96.2	dark gold
B	Figure 2b	62/4.8/61	Figure 3b	91.5	91.3	grass-green
C	Figure 2c	91/5.4/101	Figure 3c	94.4	94.5	orange-pink
D	Figure 2c	119/5.8/108	Figure 3d	92.2	92.1	bright blue
E	Figure 2d	96/6.4/86	Figure 3e	95.1	95.2	purplish-pink
F	Figure 2e	95/7.2/88	Figure 3f	93.3	93.1	purple
G	Figure 2a	46/3.8/43	Figure 3g	86.8	85.6	earthy gold

**Table 3:** Potentiodynamic scan for mirror-like SS304, CSSA/Cr/mirror-like SS304, and CSSA/Cr/ Cr/sandblasted SS304 specimens in 1 M NaCl solution.

	*ϕ*_corr_ (mV)	ⅈ_corr_ (μA/cm^2^)	Corrosion Rate (×10^−3^ mmpy)
Y0	−445.16	0.58	4.398
Y2	−161.12	0.33	2.539
Y3	−4.65	0.24	1.812

## Data Availability

The original contributions presented in the study are included in the article, further inquiries can be directed to the corresponding authors.
